# Analysis of Factors That May Affect the Effect of Atropine 0.01% on Myopia Control

**DOI:** 10.3389/fphar.2020.01081

**Published:** 2020-09-09

**Authors:** Xiaoyu Zhang, Yuliang Wang, Xingtao Zhou, Xiaomei Qu

**Affiliations:** ^1^Key NHC Key Laboratory of Myopia (Fudan University), Laboratory of Myopia, Chinese Academy of Medical Sciences, Shanghai, China; ^2^Department of Ophthalmology, The Eye and ENT Hospital of Fudan University, Shanghai, China; ^3^Shanghai Research Center of Ophthalmology and Optometry, Shanghai, China

**Keywords:** myopia, atropine, retrospective study, Chinese children, myopia progression

## Abstract

Children respond differently to atropine treatment, and predicting patient factors associated with better myopia control is important. Therefore, we aimed to evaluate factors related to myopia progression in Chinese children treated with atropine 0.01%. This retrospective study included 133 children who were administered atropine 0.01% eyedrops every night for 1 year. Enrolled children were examined at follow-up visits at 3 and 6 months, and 1 year. The primary outcome was clinically significant myopia progression (over a -0.75 diopter (D) increase in spherical equivalent (SE)). Multivariate logistic analysis was used to identify predictive factors for myopia progression. The mean baseline SE was -3.92 ± 2.76D, and the average increase in SE and axial length at 1 year from baseline were -0.55 ± 0.57D and 0.43 ± 0.52 mm, respectively. The risk of myopia progression significantly increased in children whose mothers had moderate myopia of less than -6D compared to that in children whose mothers had no history of myopia (odd ratio [OR] = 2.76, 95% confidence interval [CI]: 1.06 to 7.19, P = 0.0382). Birth by cesarean section was also a risk factor for myopia progression (odd ratio [OR] = 2.35, 95% CI: 1.30 to 4.27, P = 0.0048). The correlation between SE and treatment efficiency was linear, and the risk of myopia progression significantly decreased with increasing SE. Atropine 0.01% controlled myopia more effectively in children with higher myopia, who were delivered naturally, and whose mothers had no genetic background of myopia.

## Introduction

Myopia is a major public health concern, and refractive error is one of the five major ocular conditions considered to be an immediate priority (McCarty and Taylor, 2000; Pizzarello et al., 2004). The prevalence of high myopia has been increasing with the prevalence of myopia. Due to vision loss and complications potentially leading to blindness associated with high myopia, researchers have assessed intervention methods to slow or halt the progression of myopia (Joint World Health Organization-Brien Holden Vision Institute Global Scientific Meeting on Myopia, 2015).

Atropine 0.01% has demonstrated its efficacy in reducing axial length elongation and progression of the spherical equivalent (SE) (Chia et al., 2012; Chia et al., 2014; Chia et al., 2016; Gong et al., 2017; Yam et al., 2019a). Change in the SE of the refractive error per year is now globally accepted as a clinical marker for progressive myopia.

Previous studies have shown that children respond differently to atropine treatment. Therefore, it is difficult to predict participants who have a higher tendency of myopia progression despite atropine treatment. To clarify this, the present study was designed to evaluate factors related to myopia progression in Chinese children treated with atropine 0.01%.

## Materials and Methods

The protocol and investigators of this study were approved by the Ethics Committee of the Eye & ENT Hospital of Fudan University. Written informed consent was obtained from parents or legal guardians of the children before enrolment. The study is registered at the ClinicalTrial.gov website under the identifier ChiCTR1800017154. Our research was conducted in accordance with the tenets of the Declaration of Helsinki.

This was a retrospective clinical study designed to investigate the magnitude of myopia progression and early onset myopia in Chinese children at the age that they are most likely to experience myopia progression. We included children who received atropine 0.01% eyedrops on a treatment-as-usual, once a night basis. Participants were treated with atropine for 1 year. As atropine 0.01% is not commercially available in China, atropine 0.01% eyedrops were produced by adding 1 ml of 0.05% Kg/L atropine sulfate (atropine sulfate injection, Hubei Xinghua Pharmaceutical Co., Ltd., China) to 4 ml of polyethylene glycol eye drops (Systane ULTRA, Alcon Laboratories, Inc., USA) by the Pharmaceutical Department of Eye & ENT Hospital (Chen et al., 2019).

The inclusion criteria were as follows: 1) children aged 3-14 years; 2) baseline SE from 0 to -12.00D, astigmatism less than or equal to -2.50D; 3) eye examinations spanning across at least 12 ± 2 months of treatment; 4) no medical history predisposing severe myopia (e.g., Marfan syndrome, Stickler syndrome, and retinopathy of prematurity), abnormal ocular refractive anatomy (e.g., keratoconus, lenticonus, and spherophakia); 5) no history of other vision-threatening ocular diseases or previous intraocular surgery; and 6) no current or previous use of atropine or pirenzepine, contact lenses or other forms of treatment that may affect myopia progression.

A total of 133 children who were followed-up and reexamined for axial length as well as refractive error at 3 months, 6 months, and 1 year were enrolled; data on demographics (gender, age, parental refractive history, birth month, and method of birth), and medical and ocular treatment history were also collected. Cycloplegic refraction was used to measure the refractive errors of participants before enrollment and at the 6-month and 1-year follow-up visits. Cycloplegia was achieved with four drops of compound tropicamide eyedrops (0.5% tropicamide and 0.5% phenylephrine eyedrops; Mydrin-P, Santen Pharmaceutical, China), administered approximately 5 min apart. Cycloplegic autorefraction was performed using a desktop autorefractor (KR-8800, Topcon Corporation, Tokyo, Japan) 30 min after the last cycloplegic eyedrop was administered. Cycloplegic retinoscopy was then performed by an experienced optometrist. SE was used for statistical computations, and was calculated as the sum of the spherical power of the refraction result and half of the cylinder power.

### Outcome Measures

The efficacy of atropine was assessed by evaluating myopia progression, which is an increase of at least -0.75D from the baseline SE at 1 year after atropine 0.01% treatment. This has been defined as clinically significant worsening of myopia (based on expert opinion from the 2016 FDA Workshop on myopia progression) (FDA Dermatologic and Ophthalmic Drugs Advisory Committee, 2003. https://wayback.archiveit.org/7993/20170405133139/ https://www.fda.gov/ohrms/dockets/ac/03/briefing/3988B1_02_Novartis%20Briefing%20Document.pdf. Accessed 17 Jan 2017).

### Statistical Analysis

A stepwise, multivariate logistic regression analysis was performed using the IBM SPSS Statistics for Mac V.24.0. We compared patient demographics and other clinical characteristics between the myopia progressor and non-progressor groups. We compared the two groups using the Mann–Whitney U-test or the χ2 test. Binary logistic regression was also performed to identify the factors that increase the risk of myopia progression. Multivariate logistic regression was also conducted.

## Results

A total of 133 eligible participants were qualified for the primary analysis at 1 year. Baseline characteristics are shown in **Table 1**. The average age of participants at treatment onset was 5.79 ± 2.20 years. Seventy of the participants were male, and 63 were female. As for age, 47.37% of the participants were pre-school children (lower than 6 years old), and 52.63% were school-age children (6 years old or above). 15.79% and 27.07% of the included patients had no mother myopia background or no father myopia background, respectively. 23.31% and 17.29% had mother or father myopia of over -6D, respectively. The mean baseline SE was -3.92 ± 2.76D. Mean changes in SE at 3 months, 6 months, and 1 year from the baseline were -0.036 ± 0.45D, -0.22 ± 0.43D, and -0.55 ± 0.57D, respectively. The mean baseline axial length was 24.79 ± 1.29 mm, and the mean changes in axial length at 6 months and 1 year from baseline were 0.26 ± 0.54 mm and 0.43 ± 0.52 mm, respectively. The mean changes in axial length at 6 months for the non-progressor and progressor groups were 0.21 ± 0.57 mm and 0.38 ± 0.47 mm (P < 0.01), respectively, whereas those at 1 year were 0.34 ± 0.58 mm and 0.62 ± 0.25 mm (P = 0.025), respectively.

**Table 1:** Summary of enrolled patients (N=133).

Characteristic/Category	Mean ± SD/N (%)
Baseline age (years)	5.79 ± 2.20
Age group (years)	
<4	21 (15.79)
4 to <6	42 (31.58)
6 to <8	41 (30.83)
8 to <10	21 (15.79)
10 to 12	8 (6.02)
Sex	
Male	70 (52.63)
Female	63 (47.37)
Ophthalmic history	
Myopia	121 (90.98)
Astigmatism	109 (81.95)
Baseline SE (D)	-3.92 ± 2.76
Baseline AL (mm)	24.79 ± 1.29
Mother refractive statement	
No myopia	21 (15.79)
<-6D	81 (60.90)
≥-6D	31 (23.31)
Father refractive statement	
No myopia	36 (27.07)
<-6D	74 (55.64)
≥-6D	23 (17.29)
Change from baseline SE	
3 months	
Mean	-0.036 ± 0.45
Median	0.00
6 months	
Mean	-0.22 ± 0.43
Median	-0.25
1 year	
Mean	-0.55 ± 0.57
Median	-0.50
Mean change from baseline AL	
6 months (N=166)	0.26 ± 0.54
1 year (N=190)	0.43 ± 0.52

SD, standard deviations; SE, spherical equivalent; D, diopters; AL, axial length.

As shown in **Table 2**, there were significant differences in the baseline SE, method of birth, and maternal refractive histories (P = 0.002, 0.004, and 0.045, respectively) between the non-progressor and progressor groups. In the myopia progressor group, baseline SE was significantly lower than the non-progressor group (-3.12 ± 2.03 and -4.26 ± 2.96). However, there were no significant differences in the other variables selected as covariates for myopia progression between the two groups.

**Table 2:** Demographics of myopia progression children versus those with no progression at 1-year follow-up after 0.01% atropine applied.

Variable	Non-progressor Mean+SD/N(%)	Progressor Mean+SD/N(%)	P-value^a^
N	186 (69.92%)	80 (30.08%)	
Age started spectacles (years)	5.82 ± 2.28	5.81 ± 1.88	0.990
Age started atropine-treated (years)	7.56 ± 2.48	6.99 ± 2.19	0.077
Time between spectacles and atropine-treated (years)	1.51 ± 1.75	1.75 ± 1.82	0.330
Baseline SE (D)	-4.26 ± 2.96	-3.12 ± 2.03	0.002
Sex			0.781
Male	89 (48.11%)	37 (46.25%)	
Female	96 (51.89%)	43 (53.75%)	
Birth months			0.183
Term delivery	173 (93.51%)	78 (97.50%)	
Premature delivery	12 (6.49%)	2 (2.50%)	
Delivery way			0.004
Natural labor	88 (47.57%)	23 (28.75%)	
Cesarean	97 (52.43%)	57 (71.25%)	
Mother refractive statement			0.045
No myopia	36 (19.46%)	6 (7.50%)	
<-6D	109 (58.92%)	52 (65.00%)	
≥-6D	40 (21.62%)	22 (27.50%)	
Father refractive statement			0.699
No myopia	53 (28.65%)	19 (23.75%)	
<-6D	100 (54.05%)	47 (58.75%)	
≥-6D	32 (17.30%)	14 (17.50%)	

N, number; SD, standard deviations; SE, spherical equivalent; D, diopters.

a t-test except sex (Fisher’s exact test).

As shown in **Table 3**, results of the binary logistic regression analysis indicated that birth by cesarean section was independently associated with an elevated risk of myopia progression than birth *via* vaginal delivery; the odds ratio (OR) was 2.25 (95% confidence interval [CI]: 1.28 to 3.95, P = 0.0048). A maternal genetic background of myopia was independently associated with an increased risk of myopia progression than the absence of a maternal genetic background of myopia; the OR was 2.86 for maternal history of myopia below -6.00D (95% CI: 1.13 to 7.22, P = 0.0259) and 3.30 for maternal history of myopia above or equal to -6.00D (95% CI: 1.20 to 9.05, P = 0.0204). Other variables, including age at the time of first spectacle use, age at the onset of atropine treatment, time between spectacle use and atropine treatment, and paternal refractive history, were not associated with a higher risk of myopia progression.

**Table 3:** Analyses of risk factors for myopia progressor at 1-year follow-up after 0.01% atropine applied, based on univariate analysis.

Variable	Mean+SD/N (%)	OR (95%CI)	P-value
Age started spectacles (years)	5.82 ± 2.17	1.00 (0.89, 1.13)	0.9897
Age started atropine-treatment (years)	7.38 ± 2.40	0.90 (0.81, 1.01)	0.0779
Time between spectacles and atropine-treatment (years)	1.58 ± 1.77	1.08 (0.93, 1.25)	0.3302
Baseline SE (absolute value) (D)	3.92 ± 2.76	0.84 (0.76, 0.94)	0.0026
Birth months			
Term delivery	251 (94.72%)	1.0	–
Premature delivery	14 (5.28%)	0.37 (0.08, 1.69)	0.1996
Delivery way			
Natural labor	111 (41.89%)	1.0	–
Cesarean	154 (58.11%)	2.25 (1.28, 3.95)	0.0048
Mother refractive statement			
No myopia	42 (15.85%)	1.0	–
<-6D	161 (60.75%)	2.86 (1.13, 7.22)	0.0259
≥-6D	62 (23.40%)	3.30 (1.20, 9.05)	0.0204
Father refractive statement			
No myopia	72 (27.17%)	1.0	–
<-6D	147 (55.47%)	1.31 (0.70, 2.46)	0.3982
≥-6D	46 (17.36%)	1.22 (0.54, 2.77)	0.6332

SD, standard deviations; OR, odds ratio; CI, confidence interval; SE, spherical equivalent; D, diopters.

After adjusting for all variables listed in **Table 4**, multivariate logistic regression analysis was performed to determine the magnitude of myopia progression despite treatment with atropine 0.01%. Regarding the different birth methods, a significantly higher evaluated risk of myopia progression was associated with birth by cesarean section than with birth *via* vaginal delivery; the adjusted odds radio (aOR) was 2.35 (95% CI: 1.30 to 4.27, P = 0.0048). Regarding parental genetic background of myopia, a significantly increased risk of myopia progression was related to maternal history of myopia below -6.00D than to the absence of maternal genetic background of myopia; the aOR was 2.76 (95% CI: 1.06 to 7.19, P = 0.0382).

**Table 4:** Analyses of risk factors for myopia progressor at 1-year follow-up after 0.01% atropine applied with categorized delivery parameters and parental myopia genetic background, based on multivariate analysis.

Variable	Non-adjusted		Adjust	
	OR(95%CI)	P-value	OR(95%CI)	P-value
Birth months^a^				
Term delivery	1.0	–	1.0	–
Premature delivery	0.37 (0.08, 1.69)	0.1996	0.49 (0.10, 2.46)	0.3867
Delivery way^a^				
Natural labor	1.0	–	1.0	–
Cesarean	2.25 (1.28, 3.95)	0.0048	2.35 (1.30, 4.27)	0.0048
Mother refractive statement^b^				
No myopia	1.0	–	1.0	–
<-6D	2.86 (1.13, 7.22)	0.0259	2.76 (1.06, 7.19)	0.0382
≥-6D	3.30 (1.20, 9.05)	0.0204	2.79 (0.98, 7.95)	0.0553
Father refractive statement^b^				
No myopia	1.0	–	1.0	–
<-6D	1.31 (0.70, 2.46)	0.3982	1.08 (0.55, 2.11)	0.8286
≥-6D	1.22 (0.54, 2.77)	0.6332	0.99 (0.42, 2.36)	0.9828

a Adjust for: age started spectacles; age started atropine-treatment; baseline SE;mother refractive statement; father refractive statement.

b Adjust for: age started spectacles; age started atropine-treatment; baseline SE;birth months; delivery way.

SD, standard deviations; OR, odds ratio; CI, confidence interval; SE, spherical equivalent; D, diopters.

Smooth curve fitting was performed after adjusting for relevant confounding factors, which were sex, age at the time of first spectacle use, age at the onset of atropine treatment, birth month, method of birth, and parental history of myopia. The resultant curve demonstrated a linear association between the risk of myopia progression and baseline SE. Risk of myopia progression declined by 14% when the baseline SE increased by 1D.

## Discussion

We conducted this retrospective study to evaluate factors related to the progression of childhood myopia in Chinese children treated with atropine 0.01% eyedrops. We analyzed the independent association between predictive factors and the risk of myopia progression using multiple regression analysis after adjusting for potential confounding factors and found that the risk of myopia progression during atropine treatment was significantly increased in children whose mothers had myopia of less than -6D than in those whose mothers had no history of myopia. Birth by cesarean section was also a risk factor for myopia progression during treatment. The correlation between SE and treatment efficiency was linear, and the risk of myopia progression significantly decreased with increasing SE. Myopia control may be more effective in children with higher myopia, who were delivered *via* natural labor, and who have no maternal genetic background of myopia.

Our study recorded a -0.55D progression of myopia after atropine 0.01% treatment for 1 year, which is similar to the -0.54D progression reported by Sacchi et al. in 2019 (Sacchi et al., 2019), the -0.64D progression reported in the LAMP study (Yam et al., 2019b), and the -0.43D progression reported in the ATOM2 study (Chia et al., 2012). In the present study, the mean changes in axial length at 1 year from baseline was 0.43 ± 0.52 mm. Furthermore, the overall mean change in axial length at 1 year was 0.34 ± 0.58 mm and 0.62 ± 0.25 mm (P = 0.025) the non-progressor and progressor groups, respectively. This increase in axial length was higher than that recorded in the ATOM2 study (0.24 mm) and the LAMP study (0.35 mm) (Chia et al., 2012; Yam et al., 2019b). We noted a relatively high rate of myopia progression (30.08%) in the present study despite atropine treatment. The variation in baseline characteristics of participants may have contributed to this result. Previous studies generally included children aged 5-14 years, whereas in the present study, we included children from the age of 3 years, which lowered the mean age at enrollment to 5.79 ± 2.20 years.

Risk factors for non-responsiveness to atropine treatment have been investigated in previous studies. The atropine 1% therapy (ATOM-1) study focused on variables associated with myopia progression; their results showed that younger children with higher myopia and a parental history of myopia had a greater tendency of myopia progression despite undergoing atropine 1% treatment (Loh et al., 2015). The results of the ATOM-1 study showed that maternal or paternal history of myopia separately had no significant influence on myopia progression in atropine-treated eyes, but the risk of myopia progression was 165% higher when both parents were myopic. This result is somewhat different from the finding of the present study, in that the risk of myopia progression increased by 176% in children whose mothers had myopia of less than -6D. This indicates that maternal history of low to moderate myopia has some predictive power for myopia progression during atropine 0.01% treatment. Univariate analysis showed that maternal history of myopia above or equal to -6D significantly increased the risk of myopia progression, but no significant difference was observed after adjustment for covariates. The authors of previous studies concluded that parental history of myopia increases the risk of myopia in children (Zadnik, 1997; Pacella et al., 1999; Zadnik et al., 2015; Zhang et al., 2015). The effects of maternal and paternal histories of myopia were quantitatively the same, and parental influences were additive (Kurtz et al., 2007). For children without parental genetic backgrounds of myopia, the occurrence of myopia may be related to environment and lifestyle factors, such as less outdoor activity or more near work activity. After the onset of myopia in such children, besides the effect of the use of atropine eyedrops, vision accustomization generally improves further, which weakens the effects of environmental factors that lead to myopia progression and can explain why medication yields better outcomes. Other possibilities are speculative and future studies need to be conducted to explore the association between the parental history of myopia and myopia progression during atropine treatment.

In the present study, children who were delivered *via* natural labor had better outcomes; a 135% increased risk of myopia progression was observed in children delivered *via* cesarean section. Birth by cesarean section has never been reported as a risk factor for myopia or myopia control, rather low birth weight and premature birth have been shown to simultaneously affect the development of astigmatism or myopia (Varughese et al., 2005; Wang et al., 2013; Zhu et al., 2017). Regarding the delivery method, previous clinical investigations have mainly focused on the association between cesarean delivery and allergic disorders (Brandão et al., 2016; Chu et al., 2017; Axelsson et al., 2019). It has been hypothesized that children delivered *via* cesarean section are exposed to microbial colonization which may lead to an altered gut microbiota, and may impair the natural development of the immune system (Kaplan et al., 2011; Dominguez-Bello et al., 2016). The relationship between cesarean birth and myopia progression during atropine treatment could also be due to cesarean indication, as cesarean section, which is performed upon maternal request instead of clinical indications, is very common in China (Zhang et al., 2008). Myopic mothers are more likely to choose a cesarean section; therefore, the method of delivery is also determined by the mother’s refractive status to an extent. Regardless of whether this association between birth method and myopia progression is as a result of clinical indications or the microflora that fetuses come in contact with during the delivery procedure, it still presents a significant concern.

Baseline SE was also found to be associated with myopia progression; with every -1.0D increase in the baseline SE, the risk of myopia progression was 14% lower. A previous risk factor analysis in the ATOM-1 study yielded contrary results; the results showed that children with higher baseline myopia may still experience some myopia progression while undergoing atropine 1% treatment (Loh et al., 2015). The differences between the response profiles of participants in previous studies and those of participants in our study may be due to the lower dose of atropine used in our study. Clinical studies have shown that people with higher myopia are more likely to seek myopia control interventions. Regardless, it cannot be ruled out that participants who had higher myopia had passed the stage of rapid development of myopia. Our results also suggest that atropine indications for clinical use can be broadened.

Our study had a few limitations. Firstly, we did not include any placebo control groups. Secondly, due to the retrospective design of our study, we could not evaluate the safety of atropine, and details on the cessation of medication due to adverse reactions could not be included. Due to the relatively young age of the included children, future prospective clinical studies, with a greater focus on adverse events, need be conducted.

In conclusion, our study focused on further clarifying the variables associated with myopia progression in Chinese children treated with atropine 0.01% for myopia control. Our results show that better myopia control can be achieved in children with higher myopia, who were delivered *via* natural labor, and who had no maternal genetic background of myopia. Further research to clarify the mechanisms behind these outcomes, and to identify other strategies for myopia control are needed.

## Data Availability Statement

The raw data supporting the conclusions of this article will be made available by the authors, without undue reservation, to any qualified researcher.

## Ethics Statement

The studies involving human participants were reviewed and approved by the Ethics Committee of the Eye & ENT Hospital of Fudan University. Written informed consent to participate in this study was provided by the participants’ legal guardian/next of kin.

## Author Contributions

XYZ and XMQ were responsible for conceptualizing, designing, data collection, extraction, interpretation, manuscript drafting, statistical analysis, and conducting the study. XYZ and YLW were responsible for data collection, extraction, and critical revisions of the manuscript. XYZ and XTZ were responsible for data interpretation, manuscript drafting, supervision, and critical revisions of the manuscript for important intellectual content. This article’s contents are solely the responsibility of the authors. XMQ is the guarantor of this article and takes full responsibility for this study.

## Funding

This study was supported by the Shanghai Science Popularization Project (Grant No. 17dz2301400), the Key Laboratory of the Ministry of Health in Myopia, Fundamental Research Funds (Grant No. 2018PT32019), and the National Natural Science Foundation of China for Young Scholars (Grant No. 11702063).

## Conflict of Interest

The authors declare that the research was conducted in the absence of any commercial or financial relationships that could be construed as a potential conflict of interest.
